# Aflibercept for the Treatment of Macular Edema Secondary to Idiopathic Retinal Vasculitis, Aneurysms, and Neuroretinitis Syndrome

**DOI:** 10.7759/cureus.38154

**Published:** 2023-04-26

**Authors:** José López Fontanet, Sofía C Ayala Rodríguez, Armando L Oliver

**Affiliations:** 1 Ophthalmology, University of Puerto Rico School of Medicine, Medical Sciences Campus, San Juan, USA

**Keywords:** case report, retinal ischemia, pan-retinal photocoagulation, cystoid macular edema, irvan syndrome

## Abstract

We report a case of idiopathic retinal vasculitis, aneurysms, and neuroretinitis (IRVAN) syndrome in a patient whose cystoid macular edema (CME) was successfully treated with aflibercept and pan-retinal photocoagulation (PRP). A 56-year-old male was sent to our uveitis service for further evaluation after a fluorescein angiogram revealed symmetric retinal ischemia for 360 degrees in both eyes. A fundus examination revealed an aneurysm, neuroretinitis, and occlusive vasculitis, all consistent with a diagnosis of IRVAN syndrome. An optical coherence tomography examination revealed CME of the left eye. A chest X-ray revealed minimally prominent interstitial markings. The patient had a positive QuantiFERON-TB Gold test and was treated for tuberculosis with a one-year course of isoniazid and pyrimethamine. A further workup for other infectious and autoimmune etiologies was negative. The initial treatment consisted of bilateral PRP of the areas of peripheral ischemia, treatment for which was provided in a fragmented fashion over the course of seven months. Soon after the diagnosis, he received treatment with two intravitreal injections of aflibercept (2 mg/0.5 mL), one month apart, to the left eye. Subsequently, four months following the presentation, he developed CME in the right eye, which was treated with a single intravitreal injection of aflibercept (2 mg/0.5 mL). At his last follow-up visit, four years after the initial presentation, the patient remained asymptomatic with 20/20 visual acuity in both eyes and no evidence of CME recurrence. Our case suggests that aflibercept may serve as an adjuvant to the standard treatment with PRP, especially in cases that present with associated macular edema.

## Introduction

Idiopathic retinal vasculitis, aneurysms, and neuroretinitis (IRVAN) syndrome is a rare clinical entity of unknown etiology [1,2].​ The syndrome typically affects healthy female individuals in their youth or middle age [​1].​ This disease can be diagnosed by three major and three minor criteria: retinal vasculitis, retinal macroaneurysms, and neuroretinitis; and peripheral capillary nonperfusion, retinal neovascularization, and hyperemia of the optic disk with macular exudation [1]. The pathogenesis of IRVAN is unclear. However, it is widely accepted that is a consequence of intraocular inflammation caused by the presence of mild intraocular inflammation, vasculitis, and the formation of epiretinal membranes [3].​ Some authors have proposed that IRVAN is associated with a hypersensitivity reaction to tubercular antigens, indicating a link between IRVAN and systemic disease [4]. The literature reports that treatment outcomes for IRVAN syndrome are significantly influenced by its early diagnosis [1,5]. Treatment options include pan-retinal photocoagulation (PRP), vitrectomy, corticosteroids, and cryotherapy [1,5]. ​We hereby report the effects of treatment in a case of IRVAN syndrome on the basis of clinical and imaging findings.

## Case presentation

A 56-year-old male was sent to our uveitis service for further evaluation of his peripheral retinal ischemia and disc anomalies. His past medical history was remarkable for an appendectomy, microcytic anemia, and abnormal glucose tolerance. His family history was remarkable for diabetes mellitus and hypertension. The review of systems and past social history were unremarkable.

**Figure 1:**
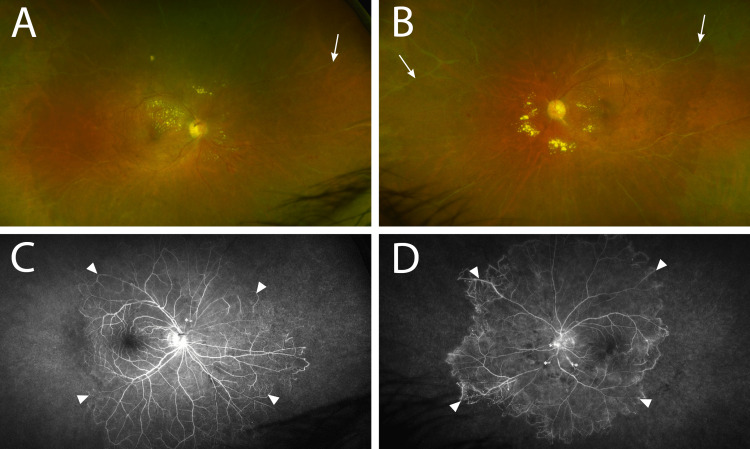
Fundus imaging at initial presentation. The right (A) and left (B) color photographs reveal multiple aneurysms (asterisk), lipid exudate, and occlusive vasculitis (arrows). The right (C) and left (D) intravitreal fluorescein angiogram reveals diffuse leakage in the choroidal watershed zone and severe marked capillary dropout for 360 degrees in both eyes (arrowheads).

**Figure 2:**
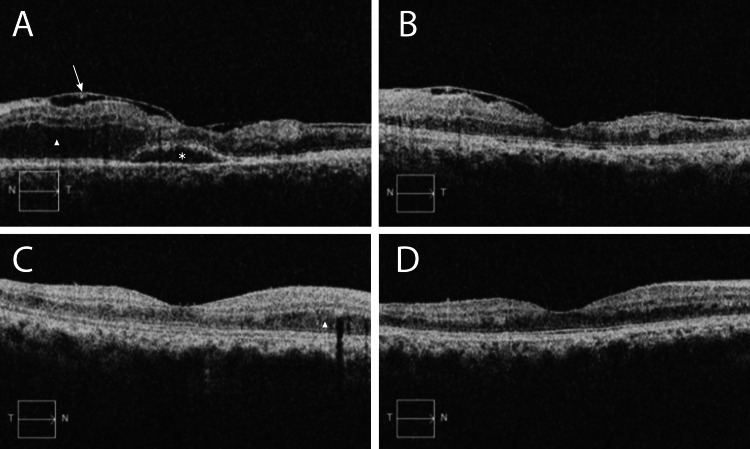
Optical coherence tomography examination of the macula. At the initial examination, the left macula revealed an epiretinal membrane (arrow) and cystoid macular edema (triangle) with subretinal fluid (asterisk). (B) The lesion resolved following two intravitreal injections of aflibercept (2 mg/0.5 mL) and showed a remarkable edema reduction. (C) After seven months of observation, the right eye showed cystoid macular edema (triangle). (D) The lesion resolved following a single intravitreal injection of aflibercept (2 mg/0.5 mL) and resulted in significant improvement of edema.

Upon ophthalmic exam, the patient’s best-corrected visual acuity was 20/20 in both eyes (OU) with a manifest refraction of +2.50 −0.75 × 100 in the right eye (OD) and +3.00 −1.00 × 085 in the left eye (OS). Intraocular pressure was 12 mmHg OU. A gonioscopy revealed open angles with no evidence of neovascularization OU. A slit-lamp examination was remarkable for nasal progressive pterygium of the conjunctiva OS and a 2+ nuclear sclerotic cataract OU. The anterior chamber was deep and clear, and there was no evidence of vitreous cells. An examination of the fundus revealed multiple aneurysms, lipid exudate, and occlusive vasculitis (Figure 1). A mild epiretinal membrane was present OU. An optical coherence tomography scan showed a posterior vitreous detachment in the OS (Figure 2). A fluorescein angiogram (FA) revealed diffuse leakage in the choroidal watershed zone and severe capillary dropout for 360 degrees OU. A Humphrey visual field (30-2) test revealed significant field constriction OU.

A comprehensive work-up revealed a positive QuantiFERON-TB Gold test; the patient was treated for tuberculosis with a one-year course of isoniazid and pyrimethamine. A chest X-ray revealed minimally prominent interstitial markings. His complete blood count and mean corpuscular volume were remarkable for the presence of microcytosis and a mildly decreased percentage of lymphocytes, respectively. Hemoglobin electrophoresis was normal and a sickle cell screening assay was negative. A C-reactive protein test, glucose tolerance test, urinalysis, and lipid panel were all within normal limits. A comprehensive metabolic panel revealed a fasting blood sugar of 100 mg/dL and was otherwise within normal limits; HbA1c was 5.8%. Studies testing for antinuclear antibodies, antineutrophil cytoplasmic antibodies, human immunodeficiency virus, syphilis, Zika, and Lyme disease were negative.

Based on these results, the uveitis specialist established the diagnosis of IRVAN. After reaching the said diagnosis, the specialist discussed the therapeutic options with the patient, who was then treated with an intravitreal injection of aflibercept (2 mg/0.5 mL) OS. Two weeks subsequent to the intravitreal injection of aflibercept, a PRP was performed in a fragmented fashion on the peripheral areas of nonperfusion OU. The following month, the patient received a second intravitreal injection of aflibercept (2 mg/0.5 mL) OS and showed a notable reduction of edema. After six weeks, he began receiving monthly PRP laser sessions (five in total) that alternated between both eyes.

After being in continuous follow-up for seven months, the patient developed cystoid macular edema (CME) superonasal to the fovea OD, which was treated with a single intravitreal injection of aflibercept (2 mg/0.5 mL) and resulted in significant improvement of his edema. A FA revealed stable ischemia and the resolution of the disc and macular leakage. Four years after the initial presentation, the patient’s symptoms resolved, his vision remains 20/20 OU, and there is no clinical evidence of CME recurrence.

## Discussion

The etiology of IRVAN remains idiopathic. However, inflammation appears to be integral to its pathophysiology. Retinal vasculitis, aneurysmal dilations, and neuroretinitis are the three primary criteria of IRVAN, while peripheral capillary nonperfusion, retinal neovascularization, and macular exudation comprise the minor criteria [2].​ A literature review of IRVAN found that blurred vision and vision loss are the most common symptoms at presentation [6]. There are two main characteristics of IRVAN syndrome, i.e., exudative retinopathy and peripheral capillary nonperfusion, that pose the greatest risk to vision [6].

The inflammatory reactions that occur in IRVAN lead to focal ischemia of the blood vessel wall, thus causing increased vessel permeability and intimal collagen remodeling. This reaction makes IRVAN syndrome susceptible to aneurysmal artery dilatation. The aneurysmal dilatation is fragile and may be prone to rupture, which would therefore provoke hemorrhage and possible exudation of the vessel’s contents throughout one or more layers of the retina, leading to visual loss [7].​ The effectiveness of intravitreal injections of aflibercept in treating retinal arterial macroaneurysms has been noted by several authors [8].​ Anti-vascular endothelial growth factor (anti-VEGF) reduces nitric oxide levels, the reduction of which leads to vasoconstriction and the decreased permeability of the vessels, thereby reducing leakage and edema [9,10].

Peripheral ischemia due to capillary nonperfusion can also promote the production of VEGF, which increases vascular permeability, resulting in macular edema and induced neovascularization [11].​ An abnormal branching or dilation of new or existing vessels near an area of nonperfusion could indicate these intraretinal microvascular abnormalities [12].​ These further complications are associated with poorer vision due to an increment in edema. Conversely, anti-VEGF blockade might reverse capillary nonperfusion and decrease neovascularization [13].

Aflibercept has some advantages that have been hypothesized to contribute to its increased efficacy since, compared to other anti-VEFG medications, it is an intermediate-sized molecule [13,14].​ Its increased penetrance is attributable to its ability to move more readily through target tissues [13]. Aflibercept has a longer intravitreal half-life than other medications, and it binds to all the isoforms of anti-VEGF-A and anti-VEGF-B, in addition to those of the placental growth factor [13-16]. This reduces the need for frequent repetitions of treatment and prolongs the clinical effect [13,14].​

Additionally, the standard treatment of PRP was administered to curtail the metabolic demand of the areas of ischemia in the retina since various articles advised the use of PRP to prevent neovascularization and resolve retinal aneurysms and leaks [6,17-19].​ Therefore, to minimize the inflammation provoked by the procedure, the patient’s treatment with PRP took place in a fragmented fashion. However, the focal laser’s effect could not be ruled out as a possible confounding effect of the edema reduction.

These findings are in concordance with those of the study of Mansour et al., in which management with intravitreal injections of aflibercept plus heavy PRP was associated with a lower rate of treatment failure compared to management with a single treatment [6]. Similarly, this form of management resulted in the resolution of retinal aneurysms and leakage, as well as a significant reduction of CME. Therefore, the use of aflibercept may be an effective alternative to conventional PRP treatment when there is an insufficient response.

## Conclusions

Our case suggests that aflibercept may serve as an adjuvant to the standard treatment with PRP, especially in cases in which macular edema is present. Despite our patient’s successful response to the described treatment, prospective studies are needed to assess its effectiveness and safety.
